# Transcriptome analysis of *Pueraria candollei* var. *mirifica* for gene discovery in the biosyntheses of isoflavones and miroestrol

**DOI:** 10.1186/s12870-019-2205-0

**Published:** 2019-12-26

**Authors:** Nithiwat Suntichaikamolkul, Kittitya Tantisuwanichkul, Pinidphon Prombutara, Khwanlada Kobtrakul, Julie Zumsteg, Siriporn Wannachart, Hubert Schaller, Mami Yamazaki, Kazuki Saito, Wanchai De-eknamkul, Sornkanok Vimolmangkang, Supaart Sirikantaramas

**Affiliations:** 1 Department of Biochemistry, Faculty of Science, Chulalongkorn University, Bangkok, Thailand; 2 Omics Sciences and Bioinformatics Center, Chulalongkorn University, Bangkok, Thailand; 3 Graduate Program in Pharmaceutical Science and Technology, Faculty of Pharmaceutical Sciences, Chulalongkorn University, Bangkok, Thailand; 4 Institut de Biologie Moléculaire des Plantes, CNRS, Université de Strasbourg, Strasbourg, France; 5 Department of Animal Science, Faculty of Agriculture at Kamphaeng Saen, Kasetsart University, Nakhon Pathom, Thailand; 6 Laboratory of Molecular Biology and Biotechnology, Graduate School of Pharmaceutical Sciences, Chiba University, Chiba, Japan; 7 Natural Product Biotechnology Research Unit, Department of Pharmacognosy and Pharmaceutical Botany, Faculty of Pharmaceutical Sciences, Chulalongkorn University, Bangkok, Thailand

**Keywords:** *Pueraria candollei* var. *mirifica*, White Kwao Krua, Miroestrol, Isoflavones, Transcriptome

## Abstract

**Background:**

*Pueraria candollei* var. *mirifica*, a Thai medicinal plant used traditionally as a rejuvenating herb, is known as a rich source of phytoestrogens, including isoflavonoids and the highly estrogenic miroestrol and deoxymiroestrol. Although these active constituents in *P. candollei* var. *mirifica* have been known for some time, actual knowledge regarding their biosynthetic genes remains unknown.

**Results:**

Miroestrol biosynthesis was reconsidered and the most plausible mechanism starting from the isoflavonoid daidzein was proposed. A de novo transcriptome analysis was conducted using combined *P. candollei* var. *mirifica* tissues of young leaves, mature leaves, tuberous cortices, and cortex-excised tubers. A total of 166,923 contigs was assembled for functional annotation using protein databases and as a library for identification of genes that are potentially involved in the biosynthesis of isoflavonoids and miroestrol. Twenty-one differentially expressed genes from four separate libraries were identified as candidates involved in these biosynthetic pathways, and their respective expressions were validated by quantitative real-time reverse transcription polymerase chain reaction. Notably, isoflavonoid and miroestrol profiling generated by LC-MS/MS was positively correlated with expression levels of isoflavonoid biosynthetic genes across the four types of tissues. Moreover, we identified R2R3 MYB transcription factors that may be involved in the regulation of isoflavonoid biosynthesis in *P. candollei* var. *mirifica*. To confirm the function of a key-isoflavone biosynthetic gene, *P. candollei* var. *mirifica* isoflavone synthase identified in our library was transiently co-expressed with an Arabidopsis MYB12 transcription factor (*At*MYB12) in *Nicotiana benthamiana* leaves. Remarkably, the combined expression of these proteins led to the production of the isoflavone genistein.

**Conclusions:**

Our results provide compelling evidence regarding the integration of transcriptome and metabolome as a powerful tool for identifying biosynthetic genes and transcription factors possibly involved in the isoflavonoid and miroestrol biosyntheses in *P. candollei* var. *mirifica*.

## Background

White Kwao Krua (*Pueraria candollei* var. *mirifica*, hereafter shortened to *P. mirifica*, shown in Additional file 1: Figure S1), has been extensively used in Thai traditional medicine as a rejuvenating herb because of its numerous phytoestrogenic constituents [1]. Phytoestrogens are plant-derived compounds that structurally or functionally mimic mammalian estrogen, and they have been applied to treat different forms of cancer, heart disease, menopausal symptoms, and osteoporosis [2]. Considering the numerous effects of phytoestrogens on human health, *P. mirifica* may be a promising candidate for treating various diseases and for developing novel medicinal products.

Three major types of phytoestrogens occur in *P. mirifica*: isoflavones, coumestans, and chromenes [3–5]. Isoflavones are an important type of phytoestrogens, which are biosynthesized via the phenylpropanoid pathway and occur predominantly in leguminous plants [6]. Seven isoflavones that have been identified in *P. mirifica* tubers: puerarin, daidzin, genistin, daidzein, genistein, kwakhurin, and mirificin [3, 5]*.* Four coumestans that also occur in the tuber of this plant are coumestrol, mirificoumestan, mirificoumestan hydrate, and mirificoumestan [7]. The chemical structure of chromenes has received considerable attention because of their low toxicity and broad pharmacological application as anticancer, antimicrobial, and anti-inflammatory agents [8]. Miroestrol and its precursor deoxymiroestrol are typically accumulated at very low levels [9], however, these compounds are the predominant chromenes in the tuberous cortex of *P. mirifica* [10], and both compounds exhibit considerably highest estrogenic activity [11]. Since both chromenes have not been reported in other plant species, their biosynthesis is likely unique to *P. mirifica*. Interestingly, although miroestrol has been identified almost six decades ago, its biosynthetic pathway and the enzymes involved are still unknown.

**Fig. 1:**
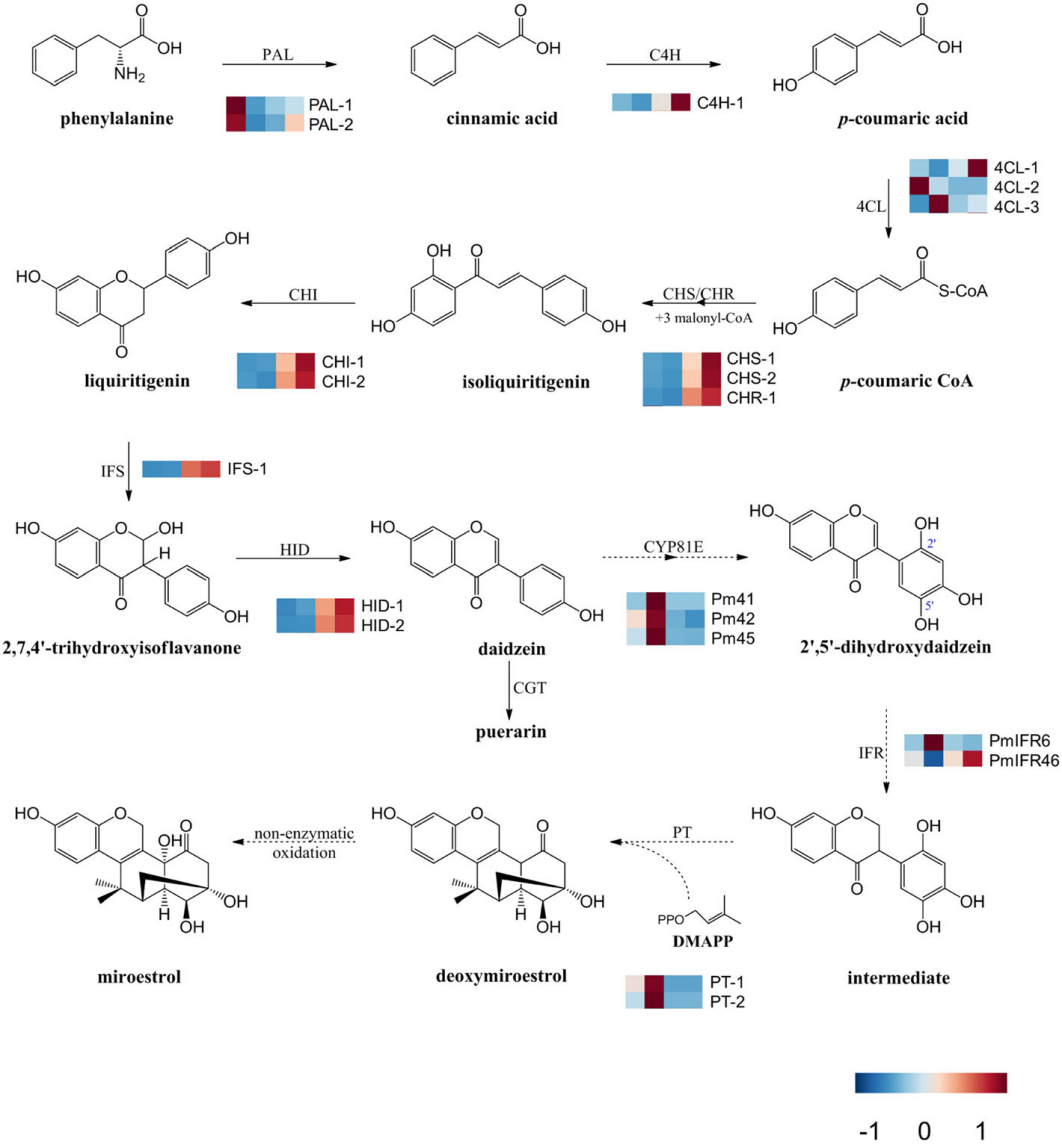
Proposed miroestrol biosynthetic pathway in *P. mirifica.* The heatmap following each gene name indicates the relative differential expression of genes in young leaves, mature leaves, tubers without cortices, and cortices of *P. mirifica*, respectively. Enzyme abbreviation: PAL; Phenylalanine ammonia-lyase, C4H; Cinnamate 4-monooxygenase, 4CL; Coumarate-CoA ligase, CHS; Chalcone synthase, CHI; Chalcone isomerase, IFS; 2-hydroxyisoflavone synthase, HID; 2-hydroxyisoflavone dehydratase, CGT; *C*-glycosyltransferase, CYP81E; Cytochrome P450 subfamily 81E, IFR; Isoflavone reductase, PT; Prenyltransferase. Although the assembled unigenes are hypothetical candidate genes, a comparative transcriptome analysis using *P. lobata* is a promising way to study and identify species-specific and evolutionary conserved pathways involved in the highly complex biosynthesis of miroestrol

Regarding biosynthesis of phytoestrogens, the early steps of isoflavonoid and flavonoid are generally similar, starting with phenylalanine ammonia lyase removing amides from the first substrate, phenylalanine, to produce cinnamic acid that is hydroxylated by cinnamate 4-hydroxylase to produce *p*-coumarate. The enzyme 4-coumarate-CoA ligase then activates *p*-coumarate by attaching a co-enzyme A (coA), and subsequently, chalcone synthase (CHS) binds *p*-coumaroyl-CoA to three molecules of malonyl-CoA to form a chalcone skeleton. Chalcone can be converted to flavanone by chalcone isomerase. Liquiritigenin is a substrate for both the flavonoid and the isoflavonoid pathway. Isoflavonoids are generally synthesized from common intermediates (either liquiritigenin or naringenin) within the recognized flavonoid biosynthetic pathway by aryl migration, which is catalyzed by isoflavone synthase (IFS). This pathway leads to the formation of an intermediate product, 2-hydroxyisoflavanone, which is then dehydrated to daidzein through catalysis by 2-hydroxyisoflavanone dehydratase (Fig. 1) [12]. CHS and IFS have been identified in *P. mirifica* several years ago [13, 14], however, their enzymatic functions have not been elucidated. Udomsuk et al. [15] suggested that miroestrol biosynthesis may share a common pathway with isoflavonoid biosynthesis due to their similar backbone structure. So far, no biosynthetic genes or enzymes involved in miroestrol biosynthesis have been reported, thus also the transcription factors responsible for regulating the expression of these biosynthetic genes remain to be identified. V-myb myeloblastosis viral oncogene homolog transcription factors (MYB TFs), which are the largest plant transcription factor family, have been reported to possess key functions in regulating the synthesis of phenylpropanoid-derived compounds in plants [16]. These proteins have attracted substantial interest regarding phytoestrogen biosynthesis in plants.

Transcriptomes produced from high-throughput sequencing of various plants are a potential source for identifying genes involved in the biosynthesis of different secondary metabolites. The transcriptome of *Pueraria lobata*, a species closely related to *P. mirifica,* has been published previously [17, 18]. Although these two plants produce similar types of isoflavones, miroestrol occurs only *in P. mirifica*. In the afore-mentioned studies, *P. lobata* genes that encode core isoflavone biosynthetic enzymes were identified, and their expression levels in various tissues were examined.

In the current study, we high-throughput sequenced *P. mirifica* to produce transcriptome libraries of young leaves, mature leaves, cortex-excised tubers, and tuberous cortices, and we de novo assembled the transcriptomes to characterize the biosynthetic pathway of miroestrol. Additionally, MYB TFs involved in isoflavonoid biosynthesis were also identified. This integrative approach of using transcriptomics and metabolomics provides new insights for the prediction and identification of putative biosynthetic genes and transcription factors that are potentially involved in *P. mirifica* isoflavonoid and miroestrol biosynthetic pathways.

## Results

### De novo transcriptome assembly of *P. mirifica*

**Fig. 2:**
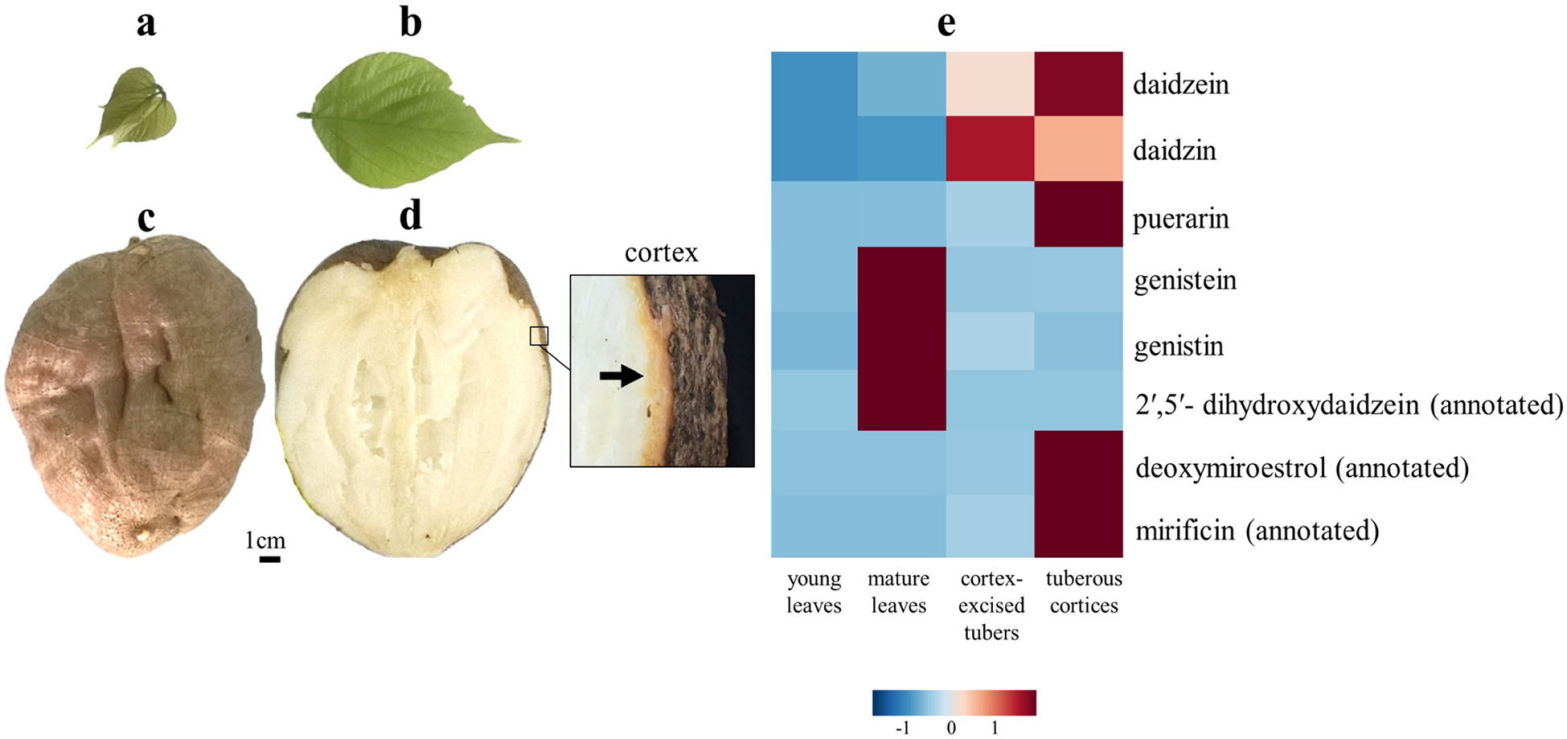
Young leaves (**a**), mature leaves (**b**) and tuber (**c**) morphology of *P. mirifica* cultivar SARDI19 used for transcriptome assembly, RNA-Seq, qRT-PCR, and HPLC-QTOF-MS/MS analysis (left). Cross section of three-year old whole tuber (**d**). Accumulation of isoflavonoids and chromenes in four different tissues of *P. mirifica* as measured with HPLC-QTOF-MS/MS (**e**). The analysis was performed with three biological replicates

To obtain nucleotide sequences of expressed genes in various tissues of *P. mirifica*, we constructed a cDNA library from pooled tissues including, young leaves, mature leaves, cortex-excised tubers, and tuberous cortices (Fig. 2a-d). The library was processed using an Illumina Hiseq 2000 platform, yielding approximately 8.2 G base pairs and a total of 7,386,137,640 clean nucleotides (nt). The de novo assembly resulted in 166,923 contigs (62,567,517 nt) and 104,283 unigenes (81,810,584 nt), the mean length/N50 for contigs and unigenes was 375/734 nt and 785/1558 nt, respectively. To assess the completeness of transcriptome data, we performed a BUSCO analysis compared to the 2121 single-copy orthologs of the eudicot lineage. The de novo transcriptome assembly was complete to 87.4, 6.9% of contigs were fragmented, and 5.7% of the transcriptome was missing (Additional file 7: Table S1). In addition, the assembly produced here was compared to that of other leguminous plants (summarized in Table 1).

**Table 1:** Comparison of *P. mirifica* de novo assembly to other leguminous plants

Description	*P. mirifica*	*P. lobata* [18]	*Ammopiptanthus mongolicus* [19]	*Millettia pinnata* [20]	*Medicago sativa* [21]
Total Clean Reads (Mb)	82	73	67	80	29
Total Clean Nucleotides (Mb)	7386	6974	6056	7219	5643
Contig					
Total Number (kb)	167	336	149	109	81
Total Length (Mb)	63	390	51	40	71
Mean Length (nt)	375	1162	345	365	873
N50 (nt)	734	1988	619	682	1323
Unigene					
Total Number (kb)	104	164	85	54	40
Total Length (Mb)	82	112	57	42	33
Mean Length (nt)	785	683	675	787	803
N50 (nt)	1558	1153	1191	1204	1300

Values shown in Mb and Kb are rounded. *Mb* Megabase, *kb* Kilobase, *nt* Nucleotide.

### Functional annotation and classification of protein

Functional annotation of the assembled transcripts provides insights in potential metabolic functions and biological processes within an organism. The functional annotation of *P. mirifica* assembled transcripts was performed based on similarities with proteins or transcripts according to information that is available in various public databases. The statistics of functional annotation are summarized in Additional file 8: Table S2. Aligned unigenes showed significant homologies using the National Center for Biotechnology Information (NCBI) non-redundant protein database. Based on the BLAST similarity distribution, over 68% of unigenes exhibited similarities greater than 80%. A top-hit species distribution analysis showed unigenes with BLAST hits sharing high sequence similarities with *Glycine max* (85.3%), *Medicago truncatula* (6.2%), and *Vitis vinifera* (1.6%).

The clusters of orthologous groups (COG) indicated the functional classification of each unigene at the cellular level. In total, 1091 unigenes were predicted to be involved in secondary metabolite biosynthesis (Additional file 2: Figure S2). Gene ontology (GO), which classifies standardized gene function, is useful for annotating gene functions and gene products in various organisms. GO is based on three major dependent factor categories: biological processes, molecular functions, and cellular components. The 37,058 unigenes yielded a corresponding GO term that can be further classified into 56 sub-categories: 23 categories related to biological processes, 17 to cellular components, and 16 to molecular functions. Additional file 3: Figure S3 shows the substantial number of transcripts related to cellular components and metabolic processes. The remaining 67,225 unigenes produced no BLAST hits. These unannotated unigenes may be uncharacterized genes or assembled sequences that were too short to produce hits.

The Kyoto Encyclopedia of Genes and Genomes (KEGG) is a comprehensive database for identifying biological pathways and for functional annotation of gene products. Pathway-based annotation helps produce an overview of the different metabolic processes that are active within an organism, and it helps improve our understanding of biological functions of unigenes. All unigenes were analyzed using the KEGG pathway with an *e*-value cutoff of < 10^− 5^. We obtained 33,317 unigenes, 3997 of which were related to biosynthesis of secondary metabolites and 8103 to general metabolic pathways. The top-five ranking pathways were plant hormone signal transduction (2061 unigenes), endocytosis (1254 unigenes), RNA transportation (1232 unigenes), glycerophospholipid metabolism (1131 unigenes), and purine metabolism (1113 unigenes). Regarding the crucial capacity of leguminous plants to accumulate functional flavonoids, 476 flavonoid biosynthetic unigenes and 167 isoflavonoid biosynthetic unigenes are shown in Additional file 4: Figure S4. These functional annotations were used for identifying genes involved in isoflavonoid biosynthesis in *P. mirifica*.

### Proposed miroestrol biosynthetic pathway, differential accumulation of transcripts associated with isoflavonoids, and miroestrol biosynthesis

Miroestrol biosynthesis potentially shares a pathway with isoflavonoid biosynthesis [15]. We propose that daidzein, a common isoflavone aglycone, is hydroxylated by at least two cytochrome P450 enzymes at the 2′ and 3′ carbon of the B-ring to produce 2′,5′-dihydroxydaidzein; these enzyme may be members of the CYP81E subfamily which is known to use isoflavones as substrates [22–25]. Then, 2′,5′-dihydroxydaidzein would be reduced by isoflavone reductase and subsequently would be prenylated by a prenyltransferase using dimethylallyl diphosphate as a co-substrate to produce deoxymiroestrol and miroestrol (Fig. 1).

Based on a functional annotation of each unigene found in the *P. mirifica* de novo transcriptome assembly and phylogenetic analyses, a total of 14 putative genes involved in isoflavone biosynthesis were predicted as follows: two *PAL* genes (encoding phenylalanine ammonia lyase), one *C4H* gene (encoding cinnamate 4-hydroxylase), three *4CL* genes (encoding 4-coumarate-CoA ligase), two *CHS* genes (encoding chalcone synthase), one *CHR* gene (encoding chalcone reductase), two *CHI* genes (encoding chalcone isomerase), one *IFS* gene (encoding IFS), and two *HID* genes (encoding 2-hydroxyisoflavanone dehydratase). In addition, a total of seven putative genes were identified in our proposed miroestrol biosynthetic pathway: three *CYP81E* genes, two *IFR* genes (encoding isoflavone reductase), and two *PT* genes (encoding prenyltransferase). To investigate gene expression levels of these unigenes across four tissues of *P. mirifica*, reads per kilobase million of transcripts were calculated from the RNA sequencing data generated using a NextSeq500 platform. An analysis of differentially expressed genes (DEG) on these unigenes (probability threshold q = 0.9) was visualized as a heat map based on our proposed miroestrol biosynthetic pathway (Fig. 1). Their sequences and RPKM values are shown in Additional file 9: Table S3. Additionally, a total of 16 putative genes encoding UDP-glycosyltransferase that are potentially involved in this pathway were phylogenetically identified from the transcriptome data. Their DEGs across the four tissue types are shown as a heatmap in Additional file 5: Figure S5.

**Fig. 3:**
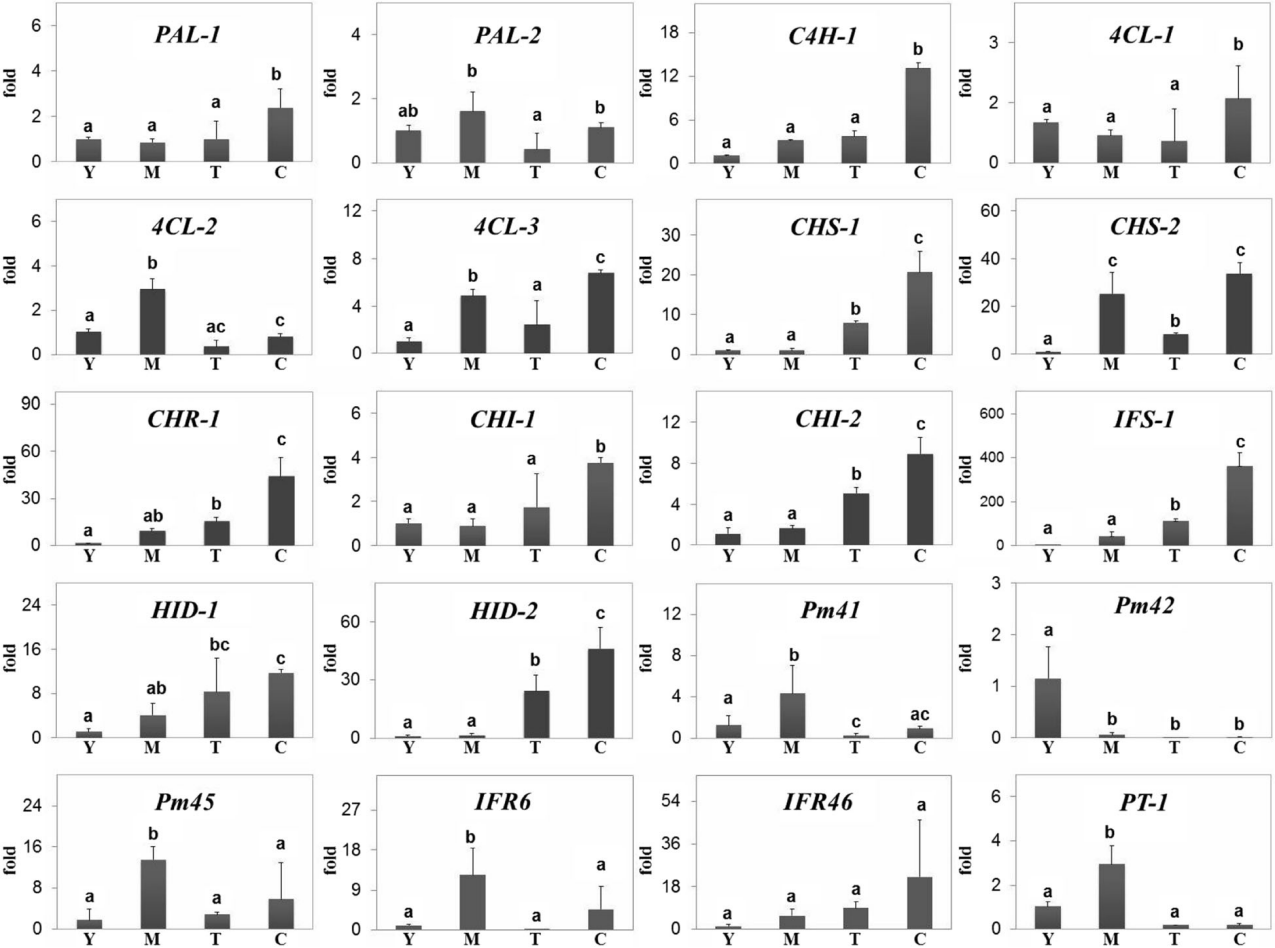
Relative expression of the candidate unigenes involved in isoflavone biosynthesis across four tissues of *P. mirifica* by qRT-PCR. Tissue abbreviations: Y; young leaves, M; mature leaves, T; cortex-excised tubers, C; tuberous cortices. Gene abbreviations: PAL; Phenylalanine ammonia-lyase, C4H; Cinnamate 4-monooxygenase, 4CL; Coumarate-CoA ligase, CHS; Chalcone synthase, CHI; Chalcone isomerase, IFS; 2-hydroxyisoflavone synthase, HID. Significantly different expression levels of putative genes (ANOVA, post-hoc Duncan test, *P* < 0.05) have different lowercase letters

To validate the DEG analysis obtained from RNA sequencing, candidate unigenes were selected and analyzed based on their expression levels in the four tissue types using quantitative real-time reverse transcription polymerase chain reaction (qRT-PCR). As expected, expression levels of the selected unigenes were positively correlated with DEGs identified by RNA sequencing (Fig. 3). Indeed, most of the isoflavone biosynthetic genes were highly expressed in tuberous cortices, compared to young leaves, mature leaves, and cortex-excised tubers; however, *Pm*CYP81Es, *Pm*IFRs, and *Pm*PT higher expressed in mature leaves than in the other tissues.

### Phytoestrogens and annotated constituents in *P. mirifica*

High-performance liquid chromatography (HPLC) coupled to tandem mass spectroscopy (MS) is a powerful tool with high selectivity and sensitivity. Seven phytoestrogens were identified in *P. mirifica* by comparison with standard compounds based on their retention times and MS fragmentation patterns. These phytoestrogens included daidzein, daidzin, genistein, genistin, 2′-hydroxydaidzein, 3′-hydroxydaidzein, and puerarin (Table 2). Due to the lack of standard compounds, mirificin, 2′,5′-dihydroxydaidzein, deoxymiroestrol, and miroestrol were predicted on the basis of mass accuracy ranges and MS fragmentation patterns searches conducted on the METLIN metabolomics database [26] or/and comparisons with published data [27]. The relative abundance of major phytoestrogens found in young leaves, mature leaves, cortex-excised tubers, and tuberous cortices of *P. mirifica* are shown as a heat map in Fig. 2e. Genistein, genistin, and annotated 2′, 5′-dihydroxydaidzein were highly accumulated in mature leaves, whereas daidzein, puerarin, annotated mirificin, and annotated deoxymiroestrol were highly accumulated in tuberous cortices, compared to the other tissues. Although miroestrol was not detected in any tissue, its precursor (annotated deoxymiroestrol) was observed only in *P. mirifica* tuberous cortex*,* which is the main accumulation site of miroestrol [10].

**Table 2:** Summary of the retention times and exact masses of identified compounds

Compound name	Chemical formula	Retention time (minute)	Exact mass
mirificin	C_26_H_28_O_13_	10.07	548.15299
daidzin	C_21_H_20_O_9_	10.16	416.11073
puerarin	C_21_H_20_O_9_	10.40	416.11073
genistin	C_21_H_20_O_10_	13.70	432.10565
3′-hydroxydaidzein	C_15_H_10_O_5_	14.75	270.05282
deoxymiroestrol	C_20_H_22_O_5_	15.03	342.14672
2′-hydroxydaidzein	C_15_H_10_O_5_	15.10	270.05282
daidzein	C_15_H_10_O_4_	16.33	254.05791
2′,5′-dihydroxydaidzein	C_15_H_10_O_5_	16.95	286.04774
genistein	C_15_H_10_O_5_	18.78	270.05282

### Isoflavone production in *N. benthamiana* leaves over-expressing *P. mirifica* isoflavone synthase

Recently, *N. benthamiana* has been used to identify transient expression of several plant genes to confirm gene functions [28]. To demonstrate that our transcriptome libraries contained candidate genes involved in *P. mirifica* phytoestrogen biosynthesis, we cloned *P. mirifica* isoflavone synthase (*PmIFS*) and conducted a functional characterization using transient (co-)expression in *N. benthamiana*, which does not produce any isoflavones. Transient expression of green fluorescent protein in five-week-old *N. benthamiana* leaves was used as a negative control. Co-expression of *PmIFS* and *Arabidopsis* R2R3 MYB12 transcription factor (*AtMYB12*), a regulator enhancing metabolic flux to flavonoid biosynthesis [29], generated two novel major peaks that were identified as genistein (Additional file 6: Figure S6), suggesting that *PmIFS* is involved in isoflavone biosynthesis.

### Identification of MYB transcription factors involved in isoflavone biosynthesis

**Fig. 4:**
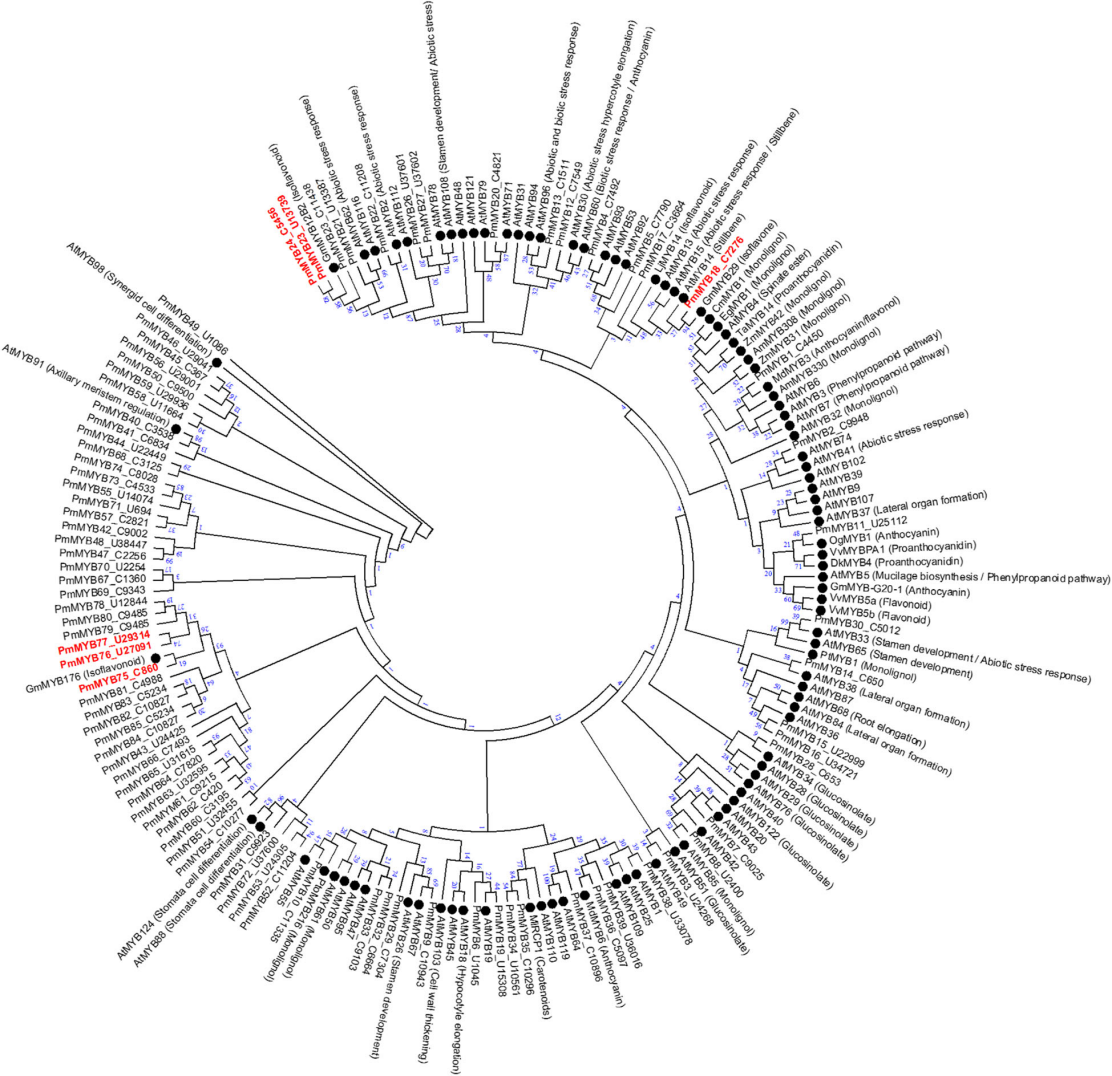
Phylogeny of MYB transcription factors associated with the phenylpropanoid pathway. The tree was constructed using the Maximum likelihood method with putative amino acid full-length MYB sequences with MEGA7. Bootstrap values are shown as percentage (100 replicates). Six P. mirifica candidate MYB transcription factors are indicated in red

**Fig. 5:**
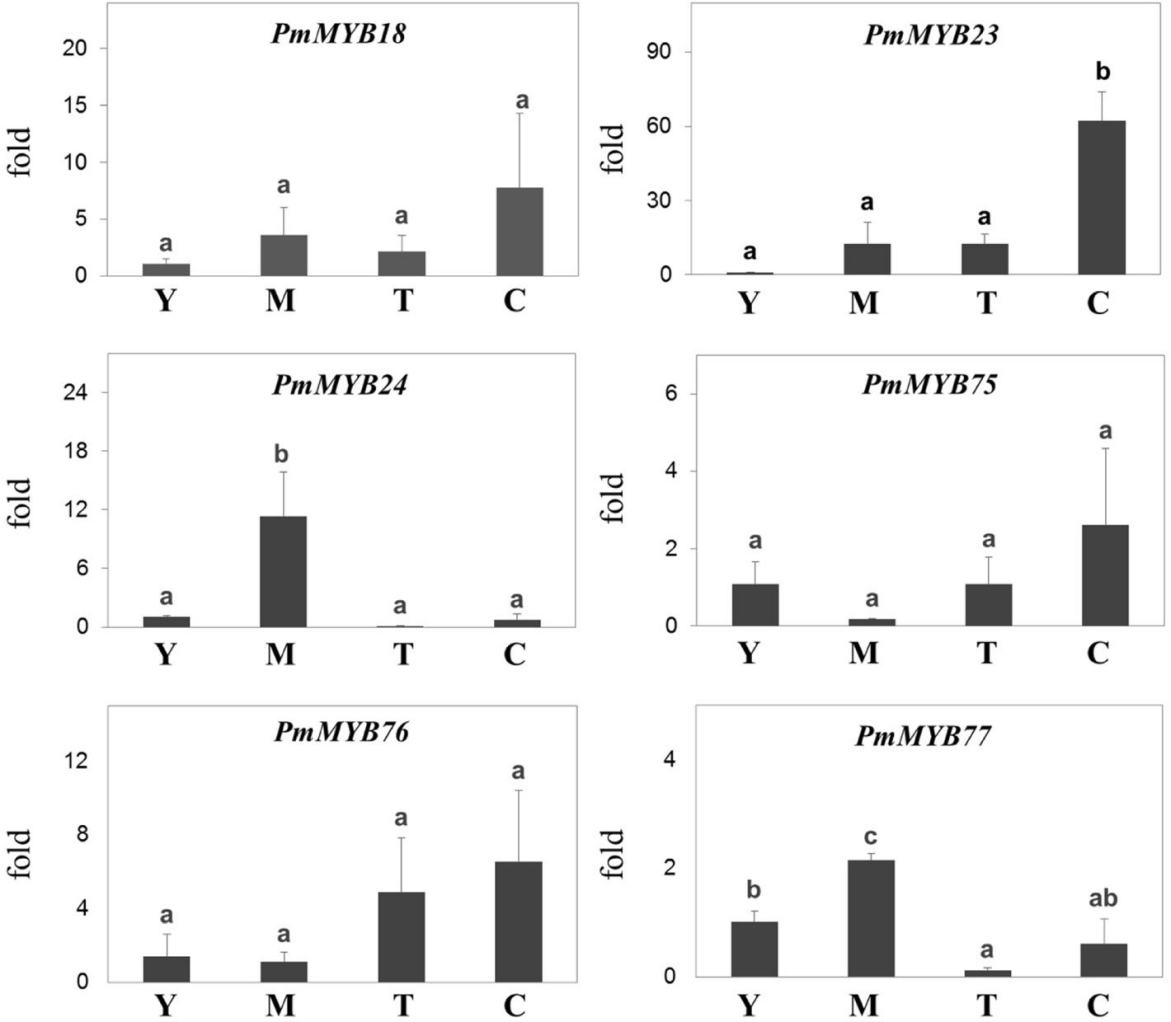
Relative expression of the candidate MYB TFs involved in isoflavone biosynthesis across four tissues of *P. mirifica* by qRT-PCR. Tissue abbreviations: Y; young leaves, M; mature leaves, T; cortex-excised tubers, C; tuberous cortices. Significantly different expression levels of putative genes (ANOVA, post-hoc Duncan test, *P* < 0.05) have different lowercase letters

We identified 85 putative genes encoding MYB transcription factors in the *P. mirifica* transcriptome. All putative *P. mirifica* MYB transcription factors (PmMYB) were aligned to known MYB transcription factors of other plants such as *Arabidopsis thaliana*, *Glycine max*, and other species, and a phylogenetic tree was produced from this alignment (Fig. 4). Based on these analyses, we found six candidate PmMYBs that are potentially involved in the regulation of isoflavonoid biosynthesis. PmMYB18 was closely related to GmMYB29, which activates expression of *IFS* and *CHS* in soybean [30]. PmMYB23 and PmMYB24 clustered with GmMYB12B2, which regulates expression of *CHS* in soybean [31, 32]. PmMYB75, PmMYB76, and PmMYB77 clustered with GmMYB176, which is also involved in controlling *CHS* expression and isoflavonoid synthesis in soybean [33, 34]. Furthermore, also *PmMYB24* and *PmMYB77* were highly expressed in mature leaves, whereas *PmMYB18*, *PmMYB23*, *PmMYB7*5, and *PmMYB76* were highly expressed in tuberous cortices (Fig. 5).

## Discussion

De novo transcriptome assembly is a useful method for gathering comprehensive information on genetic resources without the need for whole genome sequencing. In addition, this technique facilitates discovery of novel genes, molecular markers, and tissue-specific expression patterns. In the absence of comprehensive genomic data of *P. mirifica*, RNA sequencing was used to explore the *P. mirifica* transcriptome. Although the Illumina HiSeq platform has various advantages over other methods in large-scale transcriptomics research, the output data requires more time for processing and analyses. The Illumina NextSeq platform was thus designed as a faster and easier operating benchtop device to reduce costs and data processing time. Overall error rates (< 1%) are comparable across these two platforms [35]. Here, we employed an Illumina HiSeq 2000 platform to produce a transcriptome library from four tissues, and an Illumina NextSeq 500 platform to determine expression patterns of genes in each transcriptome library produced from four tissues of *P. mirifica*. The BUSCO analysis confirmed that 94.3% of the complete and fragmented sequences were included in the assembly (Additional file 7: Table S1), suggesting that the vast majority of orthologs were covered by our de novo transcriptome assembly. We produced a large dataset of unigenes from the de novo transcriptome assembly generated on the HiSeq platform. The average GC content of those unigenes was 44.56%. In eukaryotes, GC content averages approximately 20–60% [36]. The observed *P. mirifica* GC content was within this range, although slightly above those in *P. lobata* (39.9%) [17] and *Medicago truncatula* (40%) [37] but similar to that in *Glycine max* (43%) [37]. Furthermore, most of the annotated unigenes produced matches in the *Glycine max* protein database, indicating that the respective functions are conserved in these two species. Although *P. lobata* is considered closely related to *P. mirifica*, only 0.12% of the annotated unigenes matched *P. mirifica*. This is due to the limited number of full-length sequences of *P. lobata* available in the protein database, and the currently available *P. lobata* NCBI reference transcriptome is raw sequencing data [17, 18]. In addition, gene or protein names, descriptions of COG and GO terms, and possible metabolic pathways were also annotated. Notably, all candidate genes generated from the HiSeq platform data were found in the four libraries generated from the NextSeq platform data. The respective expression levels across the four tissues of *P. mirifica* can be used to reduce the number of candidates for further functional characterization.

Isoflavones are a class of flavonoids that are produced almost exclusively in leguminous plants [6]. In the *P. mirifica* transcriptome dataset, 21 putative genes involved in isoflavonoid biosynthesis were identified. The DEG values of these unigenes that were validated by qRT-PCR, exhibited high expression levels in tuberous cortices compared to those in the other three tissues. Consequently, almost all isoflavones, such as puerarin and daidzin, also showed the highest accumulation in the tuberous cortices. When comparing *P. mirifica* and *P. lobata*, gene expression and the isoflavone accumulation profile in each tissue were similar [17, 18], apart from the lack of *IFS* expression and isoflavonoid accumulation in the leaves of *P. lobata* [17]. This observation suggests a divergent evolution pattern in these two closely related species which occur in different regions (*P. lobata* in temperate zones and *P. mirifica* in tropical zones). It is possible that *P. mirifica* has evolved a mechanism to accumulate isoflavonoids in leaves and miroestrol in tuberous cortices as a means of defense. To assess whether our transcriptome data contained candidate genes, we cloned *Pm*IFS into *N. benthamiana*, which does not naturally produced isoflavonoids, for transient co-expression of *Pm*IFS and *At*MYB12, an activator of flavonoid biosynthesis [29]. This led to accumulation of genistein and genistin (Additional file 6: Figure S6). The demonstrated *in planta* function of *Pm*IFS suggests that our transcriptome libraries were suitable for identifying other genes involved in miroestrol biosynthesis. Although in a previous study, a biosynthetic pathway of miroestrol was tentatively suggested [15], the genes required for miroestrol biosynthesis remained unknown so far. Here, the biosynthetic pathway of miroestrol was reconsidered and the most plausible mechanism was proposed (Fig. 1). Seven putative genes encoding three biosynthetic enzymes involved in our proposed miroestrol biosynthetic pathway were also identified in our transcriptome data. Accumulation of deoxymiroestrol and miroestrol was reported to be higher in tuberous cortices than in cortex-excised tubers of *P. mirifica* [10]. Similarly, we detected deoxymiroestrol specifically in the tuberous cortices of *P. mirifica*; miroestrol, however, was not detected. Miroestrol can be non-enzymatically converted from deoxymiroestrol at high temperatures or in acidic or alkaline solutions during storage and extraction [38, 39]. In our experiment, *P. mirifica* tissues were harvested and rapidly frozen in liquid nitrogen, which would prevent conversion of deoxymiroestrol to miroestrol. In addition, the proposed intermediates 2′-hydroxydaidzein and 3′-hydroxydaidzein were not detected in any tissue. We hypothesized that an organized multienzyme cluster, known as metabolon, may be involved in miroestrol biosynthesis and be responsible for the absence of those cryptic biosynthetic intermediates. In fact, an increasing number of studies describe the organization of biosynthetic pathways as metabolons. Particularly, one recent study showed efficient and specific substrate transformation in a metabolon complex between IFS and chalcone reductase in soybean plants [40]. In addition, the formation of a metabolon channeling substrate towards a product without the leakage of labile intermediates has been shown [41].

Regarding putative genes involved in miroestrol biosynthesis in *P. mirifica*, we found that *CYP81E*, *IFR*, and *PT* genes were highly expressed in mature leaves, which were not the accumulation sites of miroestrol and deoxymiroestrol. Perhaps those intermediates or deoxymiroestrol are initially synthesized in mature leaves and are converted to a soluble form of glycoside that is readily transported across organs and is then stored predominantly in tuberous cortices. In fact, a glycosylated form of miroestrol has been identified in the *P. mirifica* tuber [42]. Examples of such transports are also known in tobacco, where nicotine is transported from the roots where it is biosynthesized site to the leaves where it is accumulated. Secondary metabolites are translocated between plant cells through secondary transporters and are then accumulated in the appropriate tissues or organ [43]. However, the transport of intermediates of deoxymiroestrol is still unclear and should be further investigated. Nevertheless, comprehensive functional characterization of biosynthetic genes and isotopic labeling of any intermediates is required to test this hypothesis. Alternatively, we do not exclude the possibility that deoxymiroestrol and intermediates could also be biosynthesized in the tuberous cortex, as the corresponding biosynthetic gene expression was lower there than in mature leaves. These lower expression levels of putative miroestrol biosynthetic genes could be partially responsible for the low accumulation levels of deoxymiroestrol and miroestrol. In addition, we found considerably high amounts of puerarin in tuberous cortices. Since puerarin, deoxymiroestrol, and miroestrol share an early biosynthetic pathway (Fig. 1), daidzein may be primarily diverted into the puerarin biosynthetic pathway, producing lower daidzein levels for miroestrol biosynthesis. Moreover, we phylogenetically identified a total of five putative genes annotated as *C*-glycosyltransferase, which plays a key role in producing 8-*C*-glycosylation [44] in the puerarin biosynthetic pathway. One of these putative genes, CL7002, showed 92.32% similarity to previously identified *P. lobata C*-glycosyltransferase (CGT43). These putative genes were highly expressed in tuberous cortices as compared to the other tissues (Additional file 5: Figure S5). Additionally, several glycosyltransferases have been shown to form a metabolon complex for facilitating efficient production of bioactive compounds [45–47]. These observations could contribute predominantly to daidzein utilization for producing puerarin. Suppression of *Pm*CGT may be an alternative strategy to enhance miroestrol production in tubers.

A different strategy to enhance the production of isoflavonoids and their valuable derivatives is to manipulate transcription factor regulation. In this study, we identified MYB TF, which generally acts either as a transcriptional activator or repressor for various groups of plant secondary metabolites, including isoflavonoids. Amino acid alignments and expression patterns are informative for determining the functions of individual MYB TFs. Six candidate MYBs (indicated in red in the phylogenetic tree in Fig. 4) are proposed due to their phylogenetic clustering with characterized MYB TFs that are involved in the regulation of isoflavonoid biosynthetic genes. Interestingly, four of these transcription factor genes were highly expressed in the tuberous cortex, which showed the highest accumulation of isoflavones (Fig. 5). Moreover, two of these transcription factors, *Pm*MYB18 and *Pm*MYB75, shared one branch with those MYBs reported as activators for transcription of chalcone reductase and IFS and increased isoflavonoid production [30, 34]. These two MYBs are thus important candidates for further functional characterization, and *Pm*MYB TFs may be associated with the regulation of isoflavonoid biosynthesis and miroestrol production.

## Conclusions

We identified several candidate genes encoding key enzymes or transcription factors involved in the biosynthesis of isoflavonoid and miroestrol. Integrative analyses of transcriptomics and metabolomics indicated the complexity of gene expression and metabolite profiles across tissues, suggesting that cortical tuber tissue is a major site of isoflavonoid biosynthesis. Further molecular and biochemical studies on candidate genes involved in miroestrol biosynthesis are required to identify the functions of these candidate genes.

## Methods

### Plant material, chemicals, and reagents

Leaves and tubers of an approximately two-year-old *P. mirifica* cultivar SARDI190 (Fig. 2a-d) were obtained from a *P. mirifica* farm at Kasetsart University, Kamphaeng Saen Campus, Nakhonpathom, Thailand (14°1′N, 99°58′E). The species was confirmed to be *P. mirifica* by comparison with the voucher specimens no. BCU010250 and BCU011045 kept at the Professor Kasin Suvatabhandhu Herbarium, Department of Botany, Faculty of Science, Chulalongkorn University, Thailand. Research on *P. mirifica* has been approved by the Department of Thai Traditional and Alternative Medicine, Ministry of Public Health, Thailand (DTAM1–2/2561). Seeds of *N. benthamiana* were germinated on sterilized soil in a culture room at 25 °C under a 16/8 h light/dark cycle. After germinating for five days, *N. benthamiana* seedlings were watered twice per week until harvest. Standard compounds of 2′-hydroxydaidzein [22] and 3′-hydroxydaidzein [22] were obtained from Dr. Tomoyoshi Akashi, Nihon University, Japan. Daidzein and genistein were purchased from CALBIOCHEM (Merck, Germany), and daidzin, genistin, and puerarin were purchased from Sigma-Aldrich (USA). All other chemicals were commercially available products of analytical-reagent grade.

### Construction of de novo transcriptome assembly from the combined RNA of *P. mirifica* using Illumina Hiseq 2000

Total RNA was isolated from young leaves, mature leaves, cortex-excised tubers, and tuberous cortices using the Rneasy Plant Mini Kit (Qiagen, USA), and on-column DNA was removed using the Rnase-free Dnase Set (Qiagen, USA). RNA quality was examined by agarose gel electrophoresis, and quantity was measured using a biospectrometer (Eppendorf, Germany). Aliquots of high-quality RNA of all four tissue types were pooled and subjected to Illumina Hiseq 2000 sequencing at BGI, Hongkong. Raw reads were filtered using an in-house software of BGI, filter fq, to produce clean paired-end reads and unpaired reads. Contigs and unigenes were then assembled using Trinity software [48]. We used a BUSCO (Benchmarking Single-Copy Universal Orthologs) version 3.0.2 analysis against the eudicotyledons_odb10 dataset [49, 50] to assess completeness. Raw transcriptome data were deposited in the NCBI sequence read archive (accession number: SRR6917866).

### Analysis of gene expression levels across the four tissues of *P. mirifica* generated using Illumina NextSeq500

Total RNA was extracted from each tissue type using three biological replicates. Based on spectrophotometry, high-quality RNA aliquots of the replicates were pooled per tissue type at equal quantities. The four RNA pools were then subjected to pair-end sequencing using an Illumina NextSeq500 platform. Contigs were assembled using Trinity software [48]. To estimate gene expression levels, we mapped all reads in *fastq* format to the contigs and calculated the reads per kilobase of the transcript per million mapped reads values. A non-parametric approach for identifying DEGs was used to produce four independent pairwise sample comparisons with the softawre NOISeq-sim [51]. Raw transcriptome data were deposited in the NCBI sequence read archive (accession numbers SRR10177497, SRR10177499, SRR10177500, and SRR10177498 for sequences produced from young leaves, mature leaves, cortex-excised tubers, and tuberous cortices, respectively.)

### Functional annotation of assembled sequences

The assembled sequences were first compared against the NCBI non-redundant protein database (https://www.ncbi.nlm.nih.gov/refseq/), Swiss-Prot (https://www.ebi.ac.uk/uniprot), Kyoto Encyclopedia of Genes and Genomes (KEGG; http://www.kegg.jp/kegg/), and clusters of orthologous groups (COG; *e*-value <1*E*-5; https://www.ncbi.nlm.nih.gov/COG/) by BLASTx, and nucleotide database NT (*e*-value <1*E*-5; https://www.ncbi.nlm.nih.gov/nucleotide/) using BLASTn. Then, the numbers of unigenes annotated from each database were counted. Unigenes were mapped to the COG database, potential functions were predicted, and statistical analyses were performed. BLAST2GO software v2.5.0 [52] was used to assign GO categories based on BLAST results, using default settings. All unigene GO functional categories and distribution of gene functions in different species were visualized using WEGO software [53].

### Phylogenetic analyses

All putative genes were translated to amino acid sequences using the Expasy translate tool (http://web.expasy.org/translate/). All full-length sequences of putative genes involved in isoflavonoid and chromene synthesis were aligned using BioEdit software (http://www.mbio.ncsu.edu/BioEdit/bioedit.html). We applied a maximum likelihood method with 100 bootstrap replicates using MEGA7 software [54] to produce phylogenetic trees.

### Assessment of candidate gene expression using qRT-PCR

Transcription levels of candidate genes were assessed using qRT-PCR. Total RNA isolated from the four tissue types was used (Fig. 2a-d) to synthesize single-stranded cDNA using the RevertAid First Strand cDNA Synthesis Kit (Thermo Fisher, USA). The qRT-PCR was performed using equal amounts of template DNA in a CFX96 TouchTM Real-Time PCR Detection system with iTaq Universal SYBR Green Supermix (Biorad, USA) and under the following conditions: 95 °C for 8 min followed by 40 cycles of 95 °C for 10 s, 60 °C for 15 s, and 72 °C for 30 s. The specific primers used for amplification of candidate gene sequences are shown in Additional file 10: Table S4. The *P. mirifica* unigene encoding EF1α was amplified as an internal control. Each reaction was performed using three biological replicates. The relative expression ratios of the candidate genes expressed in leaves and tuberous roots were calculated and normalized to a reference gene (EF1α) transcript level in the same sample [55].

### Metabolite extraction from *P. mirifica* samples

*P. mirifica* samples were frozen, ground to a fine powder, and lyophilized overnight. Thirty milligrams of dried powder were dissolved in 500 μL methanol (85%) to extract metabolites under vigorous shaking using a Mixer Mill MM400 (Retsch, Germany) at 25 Hz for 7 min. Crude metabolite extracts were filtered through an Acrodisc® Syringe Filter with a 0.2-μm Supor® membrane (Pall, USA) and stored at − 20 °C until analysis.

### Phytoestrogen profiling by HPLC quadrupole time of flight mass spectrometry (HPLC-QTOF-MS/MS)

The HPLC-QTOF-MS/MS analysis was performed using an Agilent HPLC 1260 series device coupled with a QTOF 6540 UHD Accurate-Mass system (Agilent Technologies, Germany). The separation of the sample solution was performed on a Luna C18 column (2) 150 × 4.6 mm, 5 μm (Phenomenex, USA). The solvent flow rate was 0.5 mL/min, with 5 μL of the sample solution being injected into the LC system. The binary gradient elution system was composed of water as solvent A and acetonitrile as solvent B, with both solvents containing 0.1% formic acid (v/v). The linear gradient elution was 5–95% for solvent B at 35 min with a post-run for 5 min. The column temperature was set at 40 °C. The conditions for the negative ESI source were as follows: drying gas (N_2_) flow rate 10 L/min, drying gas temperature 350 °C, nebulizer 30 psig, the fragmentor set to 100 V, capillary voltage 3500 V, and scan spectra from *m/z* 100–1500. The auto MS/MS for the fragmentation was set at collision energies of 10, 20, and 40 V. All data analyses were performed using Agilent MassHunter Qualitative Analysis Software B06.0 (Agilent Technologies, USA).

### Molecular cloning and transient expression of *Pm*IFS and *At*MYB12 in *N. benthamiana*

The unigene encoding isoflavone synthase (*Pm*IFS; NCBI sequence accession number MK524721) was amplified from *P. mirifica* cDNA using the following specific primers: forward 5′-ATGTTGCTGGAACTTGCAATTG-3′ and reverse 5′-TCAAGAAGGAGGTTTAGATGC-3′. For Arabidopsis MYB12 (*At*MYB12; accession number NM130314), the full-length gene sequence was amplified from *Arabidopsis* cDNA using the following gene-specific primers: forward 5′- ATGGGAAGAGCGCCATGT-3′ and reverse 5′- TCATGACAGAAGCCAAGCG-3′. The PCR was performed using 50 μL reaction volumes and a Phusion® HF high-fidelity DNA polymerase (Thermo Fisher Scientific, Finland) with the following thermocycling protocol: 98 °C for 1 min followed by 35 cycles of 98 °C for 10 s, 60 °C for 30 s, 72 °C for 1 min, and a final extension step at 72 °C for 5 min. PCR products were visualized by agarose gel electrophoresis. Amplicons of target size were ligated into a pJET1.2 vector (Fermentas, USA) for sequencing. The genes were then sub-cloned into pDONOR207 and pEAQ-HT-DEST1 [56] vectors, respectively, using a gateway cloning system according to manufacturer’s instructions (Invitrogen, USA). The recombinant pEAQ-HT-DEST1 vectors harboring *PmIFS* and pEAQ-HT-DEST1 vectors harboring *At*MYB12 were electro-transformed into *Agrobacterium tumefaciens* LBA4404. Positive clones were subjected to colony PCR. A single colony of each transformant was grown overnight (at 28 °C and under rotation at 250 rpm) in 5 mL YEB medium supplemented with 100 μg/mL rifampicin, 50 μg/mL kanamycin, and 100 μg/mL streptomycin. The cultures were then washed three times using sterilized distilled water. The resulting pellets were placed in a resuspension solution (10 mM MgCl_2_, 10 mM MES-K, and 100 μM acetosyringone, at pH 5.6). The *Agrobacterium* solution was adjusted to an A_600_ of approximately 0.4 and placed on a bench at room temperature for 2–3 h before infiltration. The *Agrobacterium* solution was infiltrated into the third and the fourth leave (from the shoot tip) of approximately four-week old *N. benthamiana* plants. After five days, the infiltrated leaves were rapidly frozen using liquid nitrogen and were subsequently ground to a powder. The powder was immediately lyophilized and was then vacuum-stored at room temperature. Thirty milligrams of powder were dissolved in 500 μL of 85% methanol, and metabolites were extracted by vigorous shaking using a Mixer Mill MM400 (Retsch, Germany) at 25 Hz for 7 min. The crude extracts were filtered through an Acrodisc® Syringe Filter with a 0.2 μm Supor® membrane (Pall, USA).

## Supplementary information

**Additional file 1: Figure S1.** Whole plant of the approximately 3-year-old *P. mirifica*. (bar = 5 cm)

**Additional file 2: Figure S2.** Clusters of orthologous groups (COG) functional classification for all assembled unigenes in *P. mirifica*. The vertical coordinates are function classes of COG, and the horizontal coordinates are numbers of unigenes.

**Additional file 3: Figure S3.** Gene ontology (GO) annotation for all assembled unigenes in *P. mirifica*. The 56 subcategories are affiliated to three main domains: biological process, cellular component, and molecular function. The GO categories were created using WEGO software.

**Additional file 4: Figure S4.** KEGG pathway enrichment analysis of assembled unigenes in *P. mirifica*. **a** The Number of unigenes in 19 sub-categories of metabolic pathway category. **b** The 21 sub-categories of metabolism of terpenoids and polyketides, and other secondary metabolites.

**Additional file 5: Figure S5.** Differential expressed genes (DEGs) predicted as UDP-glycosyltransferases that might be involved in isoflavone biosynthetic pathway across the four tissues of *P. mirifica*.

**Additional file 6: Figure S6.** Ion extract chromatogram of the annotated genistein ([M-H] = 269, RT = 18.78) of methanolic extracts of GFP transiently overexpressed in *N. benthamiana* (**a**), *P. mirifica* Isoflavone synthase (*Pm*IFS) transiently expressed in *N. benthamiana* (**b**), and *P. mirifica* Isoflavone synthase (*Pm*IFS) and Arabidopsis MYB12 transcription factor transiently co-expressed in *N. benthamiana* (**c**), as detected by HPLC-QTOF-MS/MS in negative mode.

**Additional file 7: Table S1.** Summary of the *P. mirifica* transcriptome assembly.

**Additional file 8: Table S2.** Annotation statistics.

**Additional file 9: Table S3.** Nucleotide sequences and expression profiles across the four tissues of biosynthetic genes and transcription factors involved in isoflavone and miroestrol biosyntheses.

**Additional file 10: Table S4.** DNA primer list for qRT-PCR validation.

## Data Availability

The datasets used and/or analyzed during the current study are available from the corresponding author on reasonable request.

## Abbreviations

4CL: 4-coumarate CoA ligase
BUSCO: Benchmarking Single-Copy Universal Orthologs
C: Tuberous cortices
C4H: Cinnamate 4-hydroxylase
CGT: *C*-glycosyltransferase
CHI: Chalcone isomerase
CHR: Chalcone reductase
CHS: Chalcone synthase
COG: Clusters of orthologous groups
CYP81E: Cytochrome P450 subfamily 81E
DEG: Differential expressed gene
DMAPP: Dimethylallyl pyrophosphate
EF1α: Elongation factor 1 alpha
GFP: Green fluorescent protein
GO: Gene ontology
HID: 2-hydroxyisoflavanone dehydratase
HPLC-QTOF-MS/MS: High performance liquid chromatography quadrupole time of flight tandem mass spectrometry
IFR: Isoflavone reductase
IFS: Isoflavone synthase
KEGG: Kyoto encyclopedia of genes and genomes
M: Mature leaves
MYB TF: v-myb myeloblastosis viral oncogene homolog transcription factor
nr: NCBI non-redundant protein database
PAL: Phenylalanine ammonia lyase
PT: Prenyltransferase
RPKM: Read Per Kilobase of Transcript Per Million Mapped Reads
SRA: Sequence read archive
T: Cortex-excised tubers
Y: Young leaves
