# Supplementary figures and images for: Transcriptome analysis of Pueraria candollei var. mirifica for gene discovery in the biosyntheses of isoflavones and miroestrol
Source: BMC Plant Biol. 2019 Dec 26;19:581. doi: 10.1186/s12870-019-2205-0 (PMC6933718; doi:10.1186/s12870-019-2205-0)

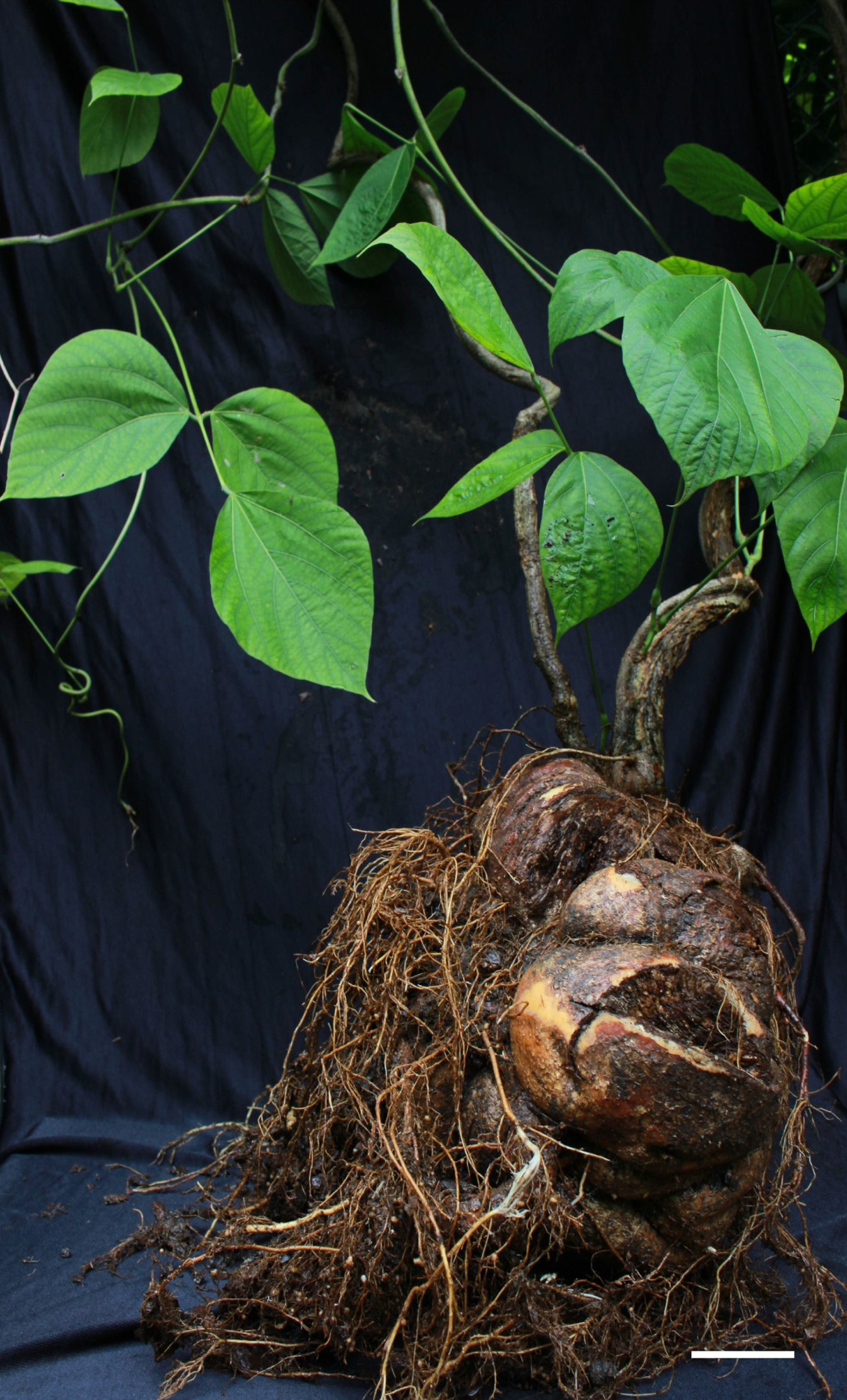

Supplement: Supplementary file 1 — Additional file 1: Figure S1. Whole plant of the approximately 3-year-old P. mirifica. (bar = 5 cm) [file 12870_2019_2205_MOESM1_ESM.tif]

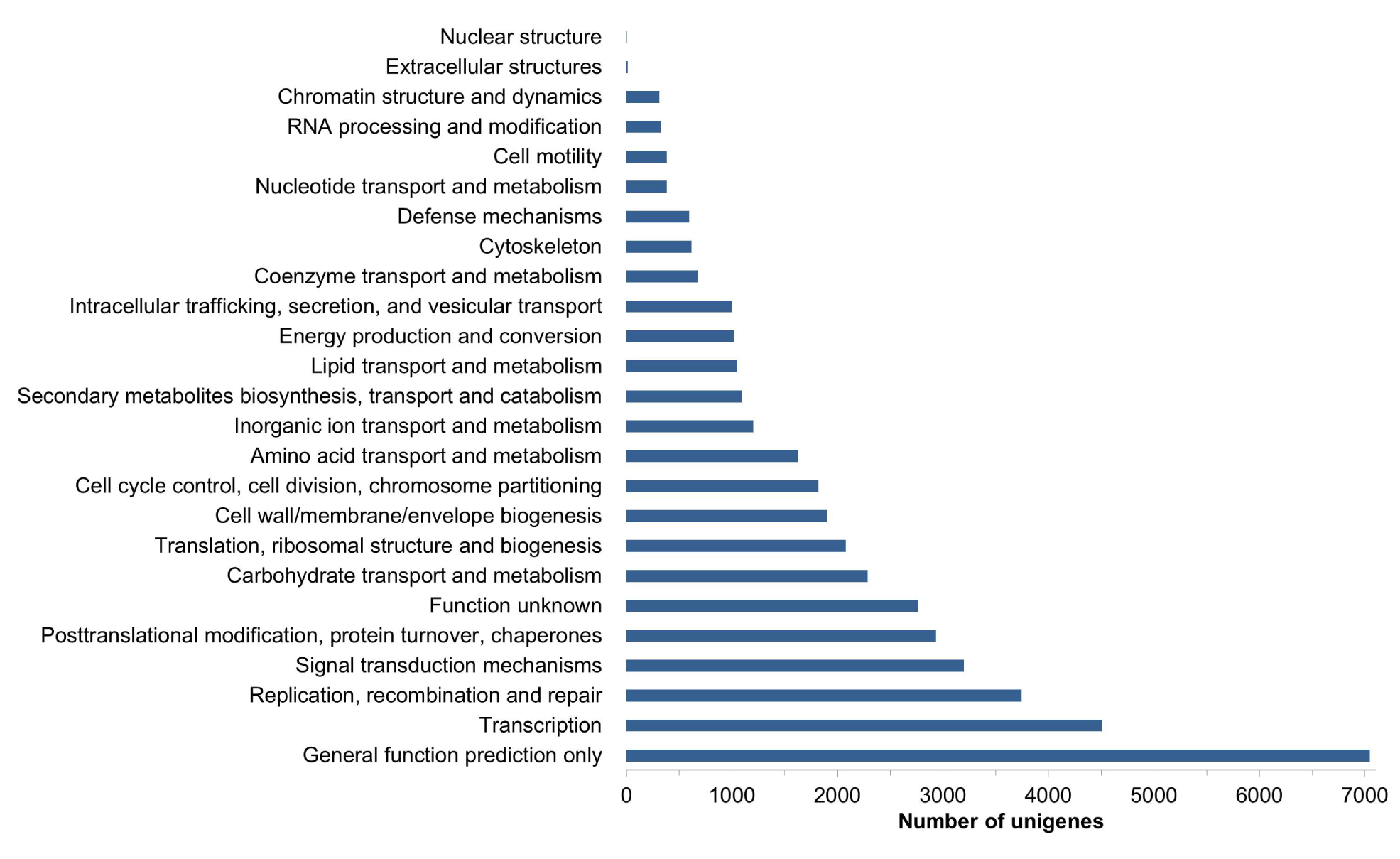

Supplement: Supplementary file 2 — Additional file 2: Figure S2. Clusters of orthologous groups (COG) functional classification for all assembled unigenes in P. mirifica. The vertical coordinates are function classes of COG, and the horizontal coordinates are numbers of unigenes. [file 12870_2019_2205_MOESM2_ESM.tif]

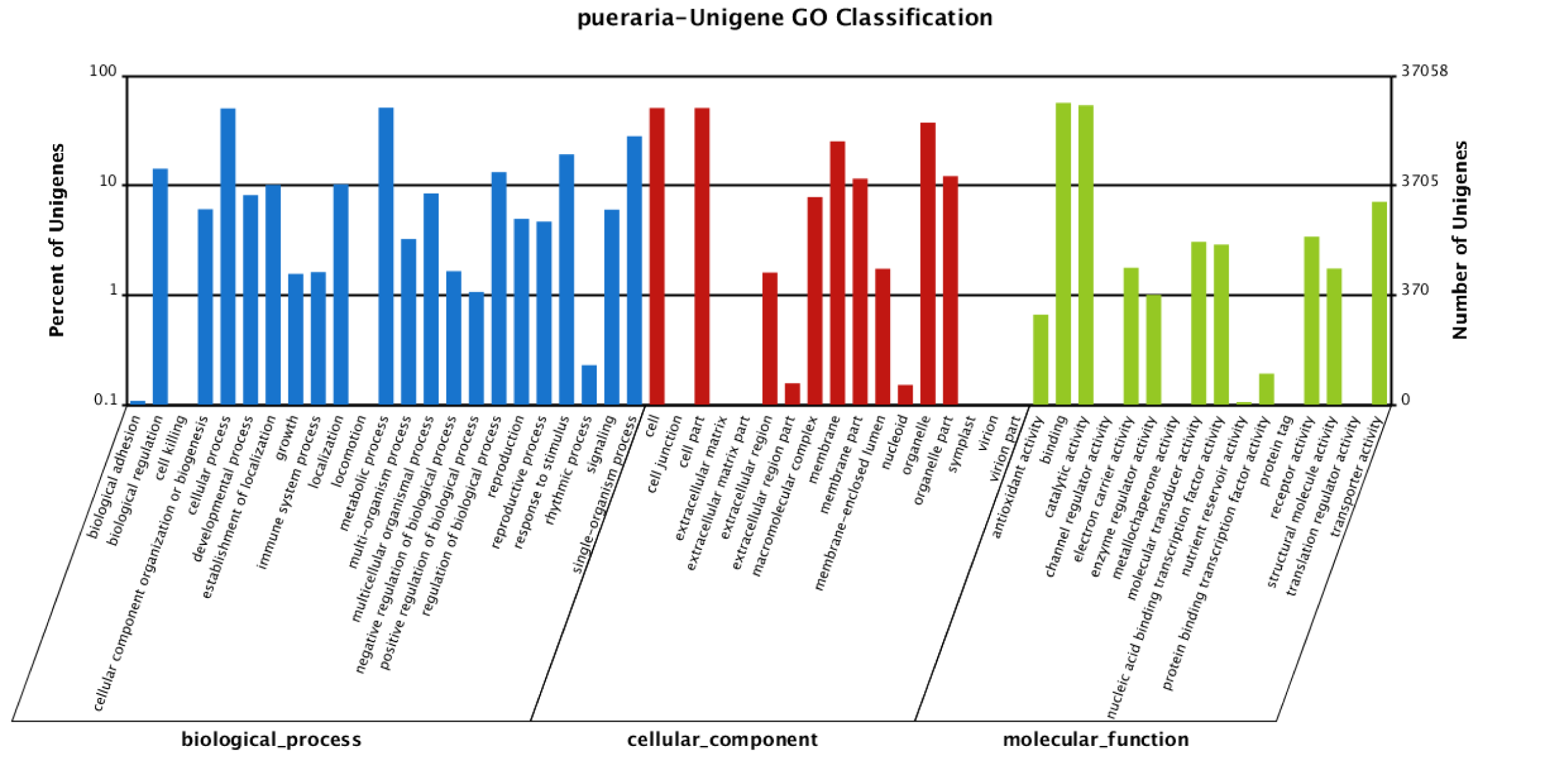

Supplement: Supplementary file 3 — Additional file 3: Figure S3. Gene ontology (GO) annotation for all assembled unigenes in P. mirifica. The 56 subcategories are affiliated to three main domains: biological process, cellular component, and molecular function. The GO categories were created using WEGO software. [file 12870_2019_2205_MOESM3_ESM.tif]

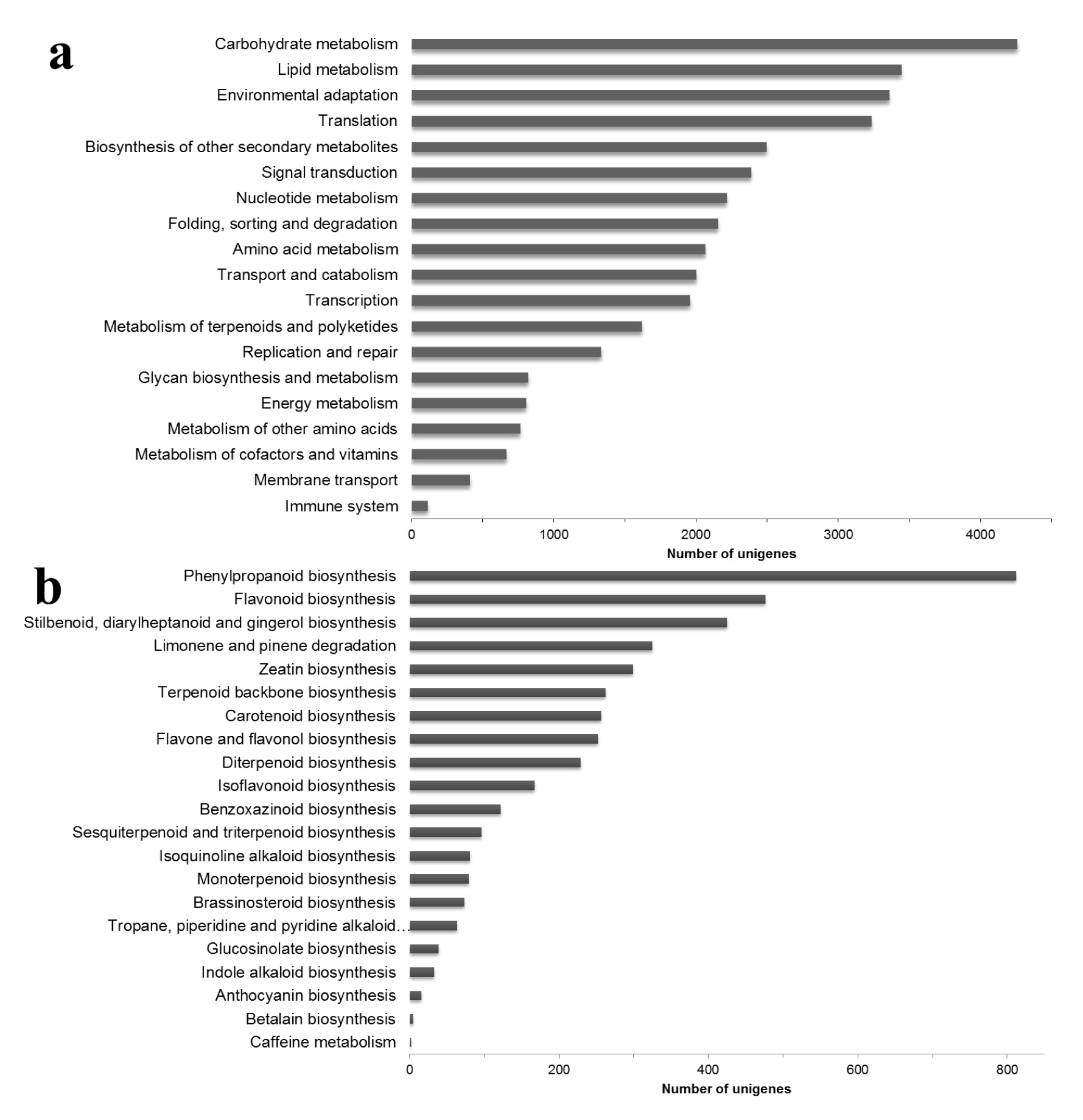

Supplement: Supplementary file 4 — Additional file 4: Figure S4. KEGG pathway enrichment analysis of assembled unigenes in P. mirifica. a The Number of unigenes in 19 sub-categories of metabolic pathway category. b The 21 sub-categories of metabolism of terpenoids and polyketides, and other secondary metabolites. [file 12870_2019_2205_MOESM4_ESM.tif]

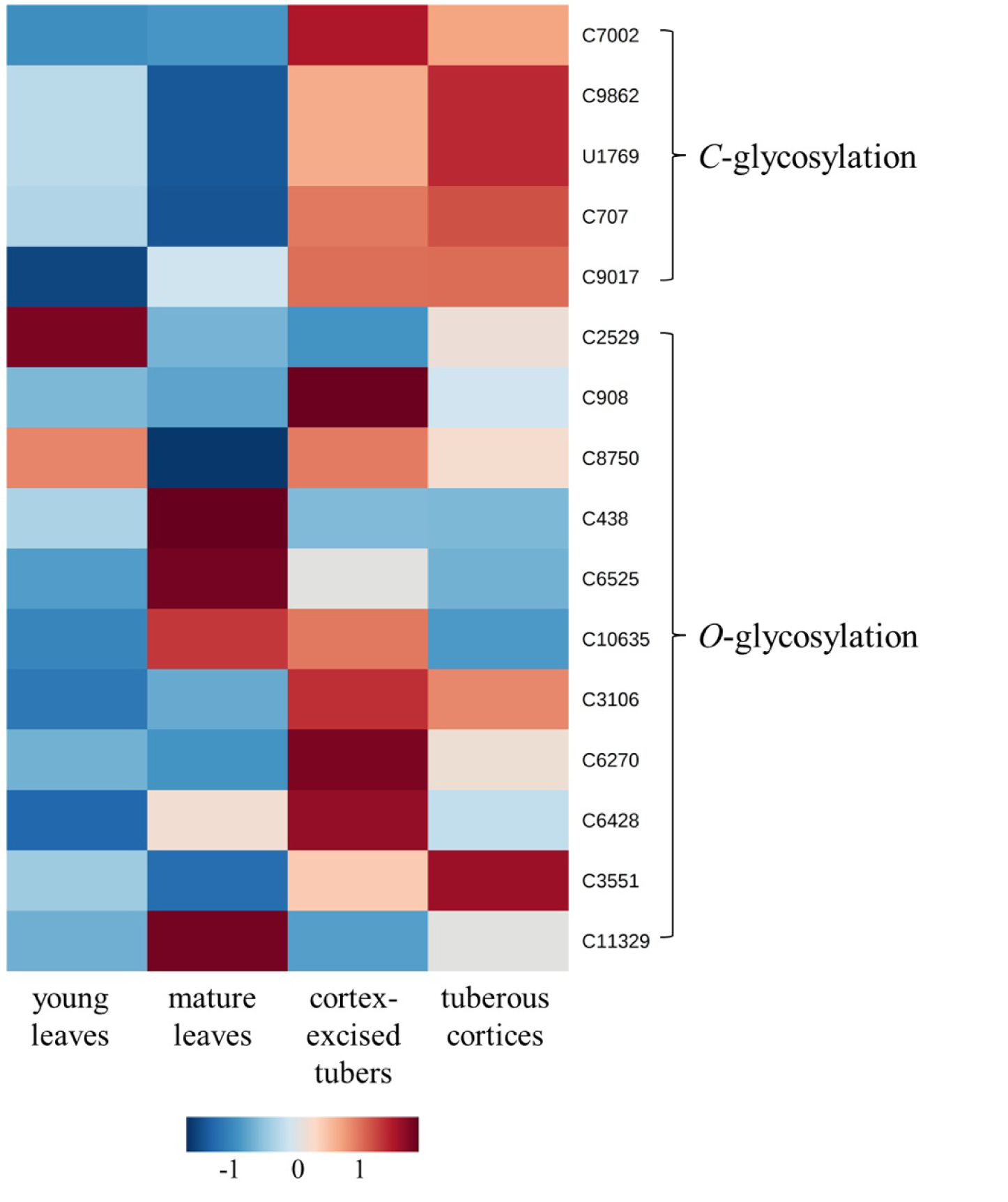

Supplement: Supplementary file 5 — Additional file 5: Figure S5. Differential expressed genes (DEGs) predicted as UDP-glycosyltransferases that might be involved in isoflavone biosynthetic pathway across the four tissues of P. mirifica. [file 12870_2019_2205_MOESM5_ESM.tif]

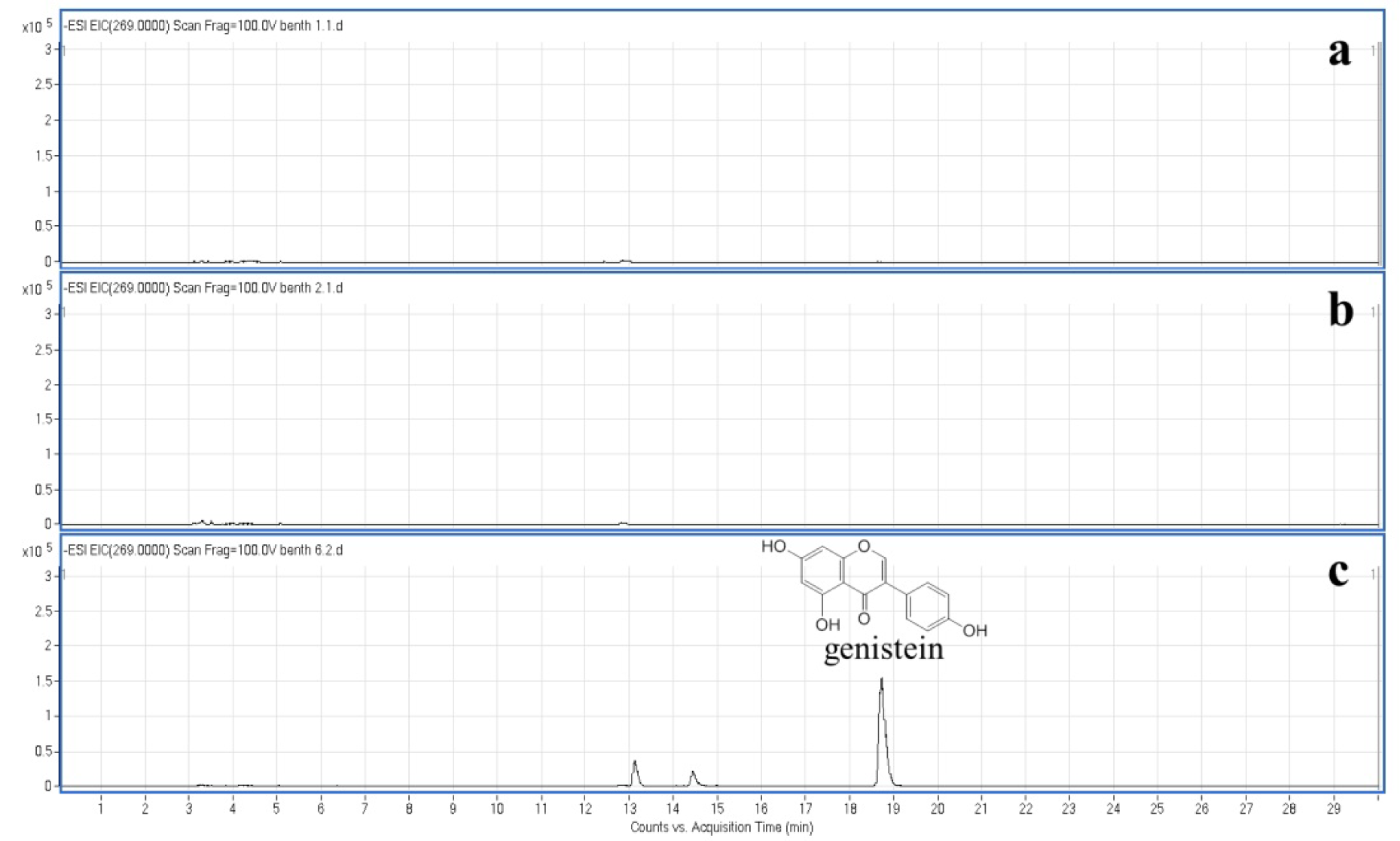

Supplement: Supplementary file 6 — Additional file 6: Figure S6. Ion extract chromatogram of the annotated genistein ([M-H] = 269, RT = 18.78) of methanolic extracts of GFP transiently overexpressed in N. benthamiana (a), P. mirifica Isoflavone synthase (PmIFS) transiently expressed in N. benthamiana (b), and P. mirifica Isoflavone synthase (PmIFS) and Arabidopsis MYB12 transcription factor transiently co-expressed in N. benthamiana (c), as detected by HPLC-QTOF-MS/MS in negative mode. [file 12870_2019_2205_MOESM6_ESM.tif]
