# Supplementary material for: Transcriptome analysis of Pueraria candollei var. mirifica for gene discovery in the biosyntheses of isoflavones and miroestrol
Source: BMC Plant Biol. 2019 Dec 26;19:581. doi: 10.1186/s12870-019-2205-0 (PMC6933718; doi:10.1186/s12870-019-2205-0)
Supplement: Supplementary file 7 — Additional file 7: Table S1. Summary of the P. mirifica transcriptome assembly. [file 12870_2019_2205_MOESM7_ESM.docx]

**Table S1.** Summary of the *P. mirifica* transcriptome assembly.

| **Descriptions** | **Results** | |
| --- | --- | --- |
| Total Raw Reads | 88,168,404 | |
| Total Clean Reads | 82,068,196 | |
| Total Clean Nucleotides (nt) | 7,386,137,640 | |
| Q20 percentage | 97.81% | |
| N percentage | 0.00% | |
| GC percentage | 44.56% | |
| **BUSCO completeness** |  | |
| Total found (Complete + Fragmented) | 2001 (94.3%) | |
| Complete BUSCOs | 1855 (87.4%) | |
| Complete and single-copy BUSCOs | 1390 (65.5%) | |
| Complete and duplicated BUSCOs | 465 (21.9%) | |
| Fragmented BUSCOs | 146 (6.9%) | |
| Missing BUSCOs | 120 (5.7%) | |
| Total BUSCO groups searched | 2121 (100%) | |
|  | Contig | Unigene |
| Total Number | 166,923 | 104,283 |
| Total Length (nt) | 62,567,517 | 81,810,584 |
| Mean Length (nt) | 375 | 785 |
| N50 (nt) | 734 | 1558 |
| Total Consensus Sequences | - | 104,283 |
| Distinct Clusters | - | 34,456 |
| Distinct Singletons | - | 69,827 |
