# Supplementary material for: Transcriptome analysis of Pueraria candollei var. mirifica for gene discovery in the biosyntheses of isoflavones and miroestrol
Source: BMC Plant Biol. 2019 Dec 26;19:581. doi: 10.1186/s12870-019-2205-0 (PMC6933718; doi:10.1186/s12870-019-2205-0)
Supplement: Supplementary file 8 — Additional file 8: Table S2. Annotation statistics. [file 12870_2019_2205_MOESM8_ESM.docx]

**Table S2.** Annotation statistics.

| **Databases** | **Number of unigenes** |
| --- | --- |
| NR | 60,302 |
| NT | 71,848 |
| Swiss-Prot | 38,416 |
| KEGG | 33,317 |
| COG | 20,247 |
| GO | 37,058 |
| **ALL** | **74,671** |
