# Supplementary material for: Transcriptome analysis of Pueraria candollei var. mirifica for gene discovery in the biosyntheses of isoflavones and miroestrol
Source: BMC Plant Biol. 2019 Dec 26;19:581. doi: 10.1186/s12870-019-2205-0 (PMC6933718; doi:10.1186/s12870-019-2205-0)
Supplement: Supplementary file 10 — Additional file 10: Table S4. DNA primer list for qRT-PCR validation. [file 12870_2019_2205_MOESM10_ESM.docx]

**Table S4.** DNA primer list for qRT-PCR validation.

| **Annotated function** | **Candidate unigene** | **5′-3′ Forward primer sequences (bp)** | **5′-3′ Reverse primer sequences (bp)** |
| --- | --- | --- | --- |
| Phenylalanine ammonialyase1 (*PAL-1*) | CL4444 | CCAGGTGAGGAATGTGATAAG (21) | CACCATTCCACTCCTTTAGAC (21) |
| Phenylalanine ammonialyase2 (*PAL-2*) | U353 | CTGAGCAACACAACCAAGA (19) | GAGGAAAGTGGAAGACATGAG (21) |
| Cinnamate-4-hydroxylase3 (*C4H-1*) | CL7513 | GAAGGTCCGTGATGAGATTG (20) | CTTGAAGGTAAGGGAGTTTGT (21) |
| Coumarate CoA ligase4 (*4CL-1*) | U33566 | GATCCTGAAACCGGTAATTCT (21) | CAGTAGCCTCTCCATCATTTAG (22) |
| Coumarate CoA ligase5 (*4CL-2*) | U32504 | TCACCGAGGATGAAATCAAG (20) | CTGAGGGTGCTTTAGGAATAG (21) |
| Coumarate CoA ligase6 (*4CL-3*) | U30063 | TCAAGCCGGTGAGATTTG (18) | TGCAACCATCCTCCTTTATC (20) |
| Chalcone synthase7 (*CHS-1*) | CL4228 | AGTGCGTGTGTGCTATTC (18) | CAGGGCCGAAACCAAATA (18) |
| Chalcone synthase8 (*CHS-2*) | U28745 | GAGGAAGAAGTCGAAGGA (19) | ACGGTCTCAACCGTAAGA (18) |
| Chalcone reductase9 (*CHR-1*) | U35087 | ATAACTGCGTTCTCTCCTCT (20) | GAGCCTCTGCAATCTCTTTC (20) |
| Chalcone isomerase10 (*CHI-1*) | U29926 | ATTTCCCACCTGGCTCTA (18) | CTCGTGTTCTGGTATTGTCTC (21) |
| Chalcone isomerase11 (*CHI-2*) | U25179 | GCTGTGTTGGAGTCAATGATA (21) | GACACCACCCTCTTTGAATAA (21) |
| Isoflavone synthase (*IFS-1*) | CL4183 | GAATGTGCCCTGGAGTTAAT (20) | TTTGGCGTCCTTACCTTTC (19) |
| Hydroxyisoflavonedehydratase (*HID-1*) | U42984 | CTGTACCACGACACTGTTAAG (21) | GGGTTGAAGAGCTGGAAAG (19) |
| Hydroxyisoflavonedehydratase (*HID-2*) | U19344 | GTTTGAGGTGGAAGAGGAAG (20) | AGCAGGAAAGAAGCCAAG (18) |
| Cytochrome P450-41 (*Pm41*) | CL7852 | GAACAAGGTTAGGGATGAGATAG(23) | CACAGGATACAGACGAAGTG (20) |
| Cytochrome P450-42 (*Pm42*) | CL5128 | ATGGAAACGAACTGGTAAGG (20) | TGACATACTTGGCACATAGC (20) |
| Cytochrome P450-45 (*Pm45*) | CL10822 | TGTCCCACGAAACACAATAG (20) | TCTCCTCTCCTTCTTCATCATA (22) |
| Isoflavone reductase (*IFR6*) | CL9120 | CACAGCAGATAAAGGGAGATG (21) | CATCGACGGTGCTGTATTT (19) |
| Isoflavone reductase (*IFR46*) | CL9537 | CCGGGATAAGGTTGTCATTC (20) | TCCACTGCTTTGATGGTATAAG (22) |
| Prenyltransferase (*PT1*) | CL740 | ATTGGTTTGTTACAGGTTTTGG (22) | CTAATGAGAGCCAGGAGAAAG (21) |
| MYB Transcription factor 18 (*PmMYB18*) | CL7276 | GAGATGCCACTTCAATACCC (20) | ATCCATGCCGTCATCAAAG (19) |
| MYB Transcription factor 23 (*PmMYB23*) | U13739 | CTCGATTCTGTGTGTCTTGG (20) | TCATCACCAGCTTCAACTAAG (21) |
| MYB Transcription factor 24 (*PmMYB24*) | CL5456 | GTAACCACGCAAAGCATTAG (20) | CAACCAACTCATCTCCCATAG (21) |
| MYB Transcription factor 75 (*PmMYB75*) | CL860 | CCTAACAGGAACAGCCTATG (20) | GAACAACCTCTCACAGTACG (20) |
| MYB Transcription factor 76 (*PmMYB75*) | U27091 | ACCGGATCCAACGAAATG (18) | GCTCTTACCGCAGCTAATAA (20) |
| MYB Transcription factor 77 (*PmMYB77*) | U29314 | CCTGTTGGTAGTTCGGATTT (20) | GCTGCTGTCTTCACCATAC (19) |
| Elongation factor 1, alpha subunit (*EF1α*) | CL2957 | GCTATGCACCTGTTCTTGAT (20) | GCTCCTTCTCAAGTTCCTTAC (21) |
